# Low Birth Weight and Related Metabolic Risk Factors, Cardio-Respiratory Fitness and Physical Activity in Adolescents

**DOI:** 10.3390/children11121523

**Published:** 2024-12-16

**Authors:** Aristides M. Machado-Rodrigues, Cristina Padez, Daniela Rodrigues, Luís P. Mascarenhas, Nuno Borges, Cátia Maia, Liliana C. Baptista, Helder Miguel Fernandes, Neiva Leite

**Affiliations:** 1 University of Coimbra, Faculty of Sport Sciences and Physical Education, 3040-248 Coimbra, Portugal; uc2018297743@student.uc.pt (N.B.); uc2017263629@student.uc.pt (C.M.); lbaptista@fcdef.uc.pt (L.C.B.); 2 University of Coimbra, Interdisciplinary Center for the Study of Human Performance (CIPER-UC), 3040-248 Coimbra, Portugal; 3 Research Centre for Anthropology and Health, University of Coimbra, 3000-456 Coimbra, Portugal; cpadez@antrop.uc.pt (C.P.); rodrigues1323@gmail.com (D.R.); 4 Department of Life Sciences, University of Portugal, 3000-456 Coimbra, Portugal; 5 UniCentro, Department of Pediatrics, Centro-Oeste State University, Irati 85040-167, PR, Brazil; lmascarenhas@unicentro.br; 6 Polytechnic Institute of Guarda, 6300-559 Guarda, Portugal; hmfernandes@ipg.pt; 7 Sport Physical Activity and Health Research & Innovation Center (SPRINT), 6300-559 Guarda, Portugal; 8 Department of Pediatrics, Federal University of Paraná, Curitiba 80060-000, PR, Brazil; neivaleite@ufpr.br

**Keywords:** adiposity, adolescence, cardiorespiratory fitness, low birth weight, metabolic syndrome

## Abstract

Background/Objectives: The associations between low birth weight (LBW) and the aggregation of metabolic risk factors (MRF) in youth remain ambiguous. Thus, this study analysed the interrelationship among MRF, LBW, and behavioural factors in adolescents. Methods: The sample of the present cross-sectional study comprised 491 youth (229 males, 262 females) aged 14–17 years. Height, weight, and BMI were assessed. Cardiorespiratory fitness (CRF) was measured using the PACER test. Physical activity (PA) was evaluated using a 3-day diary. A MRF risk score was calculated using the *Z*-scores of the five MRF criteria (HDL-C, triglycerides, BP, insulin, and blood glucose). Results: The average values for height, weight, and systolic and diastolic BP were significantly higher in males (*p* < 0.01); in contrast, females exhibited higher HDL cholesterol and insulin levels (*p* < 0.01). Boys revealed higher levels of PA (*p* < 0.01), and they achieved better CRF scores than girls (*p* < 0.01). BMI emerged as a significant predictor of clustered metabolic risk for both males (β = 0.26; 95% CI, 0.16–0.36) and females (β = 0.02; 95% CI, 0.14–0.30); additionally, the results indicated that more physically active girls had a notably lower metabolic risk (β = −0.01; 95% CI, −0.10–−0.01) than their male peers. Conclusions: LBW was found to be independently correlated with the aggregated MRF (β = −0.01; 95% CI, −0.01–0.00) among boys aged 14–17 years.

## 1. Introduction

Obesity represents a significant public health issue with a multifactorial nature, entwined with host factors (e.g., genes, epigenetics, psychological traits) and environmental factors (e.g., family, lifestyle/living conditions, policy structures, socioeconomic resources). The literature clearly supports the impact of an unhealthy lifestyle on pediatric obesity. Furthermore, obesity is positively associated with higher cardio-metabolic risk factors (CMRFs), as well as other comorbidities in youth [1,2]. Indeed, adolescents with obesity also show more CMRFs, however, not all youth exhibit the same level of risk. Perinatal factors such as birth weight could influence the likelihood of metabolic complications not just in adults [3] but also in adolescents [4,5]. Identifying sub-groups that are at risk and factors that may influence the status of CMRFs could serve a crucial role in decreasing the future incidence of cardiovascular disease.

The World Health Organization (WHO) characterizes low birth weight (LBW) as a birth weight of under 2500 g (<2499 g), irrespective of the gestation age. LBW remains a major public health issue worldwide and is linked to several both short- and long-term effects. LBW accounts for over 20 million births in the world every year and is receiving the attention of WHO. By the year 2025, the WHO projects a 30% decrease in the incidence of neonates weighing under 2500 g at birth [6]. It is well established that LBW is related to the risk of adult-onset chronic diseases [3]; however, most of the evidence is limited to outcomes in adults born with LBW. Among children and adolescents, despite being substantially less studied, birth weight (BW) has been linked with cardiovascular risk factors such as abnormal lipid levels and elevated blood pressure [4,7,8]. Indeed, there is a positive correlation between BW and the rate of weight gain after birth with adiposity in adolescents [9,10], and this adiposity serves as a significant predictor of cardiovascular risk factors. This association is somewhat attributed to changes in metabolic and cardiovascular programming resulting from restricted fetal growth which result in adipocyte hyperplasia, dysregulation of energy metabolism, and compromised vascular function [11,12]. Additionally, the transition from a hypo-trophic intrauterine environment, which can result in adipocyte hyperplasia, increases the possibility of adipose hypertrophy in a postnatal environment with access to nutrients. This mechanism can induce excessive weight gain, contributing subsequently to the development of chronic diseases, including obesity, hypertension, and heart disease [12,13].

The interaction within the uterus between the mother and child is complex, and appears to induce endogenous alterations that may disrupt organic balance and potentially lead to long-term effects by increasing the likelihood of individuals facing negative experiences in adulthood [3]. The predominant metabolic irregularities observed in adolescents diagnosed with metabolic syndrome (MetS) include an increase in waist circumference (WC; 96.2%), low HDL-C (96.2%), and hypertriglyceridemia (73.1%); furthermore, insulin resistance has similarly been observed in adolescents diagnosed with MetS [14]. This phenomenon can be attributed to environmental and behavioral variations that have contributed to an increase in sedentary lifestyles and a reduction in physical activity (PA) levels. Available data are not clear as to whether the association between BW and CMRF is independent among adolescents. Considering that differential impacts of BW on metabolic risk factors by sex were not systematically considered in the literature, this study examined the independent association between birth weight and cardio-metabolic risk factors in adolescents. It was hypothesized that adolescents classified as LBW would be more likely to present an adverse metabolic risk compared to normal birth weight youth.

## 2. Methods

### 2.1. Sample 

The present cross-sectional study was conducted in Curitiba, Paraná, Brazil, which has approximately 1,678,965 people. The city is divided into nine administrative districts that encompass a total of 293 schools. Schools were selected randomly from these districts, and every student enrolled in the selected schools was invited to take part in the study. The final sample consisted of students who provided written informed consent duly signed by their parents or guardians. An a priori sample size was computed using the following criteria: expected mean difference of 1.0, standard deviation of 2.0, power of 95%, and error (alpha two-sided) of 0.05, with Bonferroni correction. This estimate required a minimum of 123 subjects in each group. Accordingly, 491 adolescents (262 girls) aged 14 to 17 years old had completed data for metabolic variables used in the current analytical approach, and they were included in the current partial study. Among the 491 youth in the present study, approximately 80% of the boys were classified as having a normal weight, while 17% were identified as overweight and 3% were categorized as obese. For the girls, the corresponding figures were 79% for normal weight, 17% for overweight, and 4% for obesity.

This multifaceted project was approved by the Scientific Committee of the Federal University of Paraná, in which the current study is included [Study Registration: CPE 1466.131/2007-06 and CAAE: 0137.0.208.00-07 (Health National Committee: 196/96), 8 August 2007], which required anonymity and non-transmissibility of data in accordance with the principles established in the Declaration of Helsinki. Moreover, prior to data collection, informed written agreement was obtained from parents or guardians.

### 2.2. Anthropometry

Height was assessed to the nearest 0.1 cm with a portable stadiometer (Ottoboni HM-210D; Rio de Janeiro, Brazil) and weight was evaluated to the nearest 0.1 kg with a calibrated beam balance scale (Toledo 2096 PP; São Paulo, Brazil). For analysis purposes, the average of the two measurements for both height and weight were considered. All measures were objectively collected by trained research assistants at each educational institution. Students were asked to remove their shoes, and they wore t-shirts and shorts. Body mass index (kg/m^2^) was subsequently computed and labelled (i.e., normal weight and overweight/obese) using age- and sex-adjusted cut-off points [15].

### 2.3. Birth Weight

Normal BW refers to participants whose birth weight ranges from 2500 g to 3999 g. LBW was defined as under 2499 g of weight [16]. A similar procedure was used in several epidemiological studies from different geographic contexts [10].

### 2.4. Blood Sampling

Blood samples were obtained by trained research assistants (i.e., nurses) from the antecubital vein between 8:00 and 10:00 a.m. Participants were in a fasted state for at least 10 h and seated during the procedure; the samples were collected in gel vacuum tubes (Sarstedt, Sintra, Portugal). Following a resting period of approximately 30 min at room temperature, the samples underwent centrifugation for 10 min at a speed of 3000 rpm to isolate serum. Within 30 min, the samples were separated into aliquots and stored at −80 °C until further analysis. HDL-C levels, TG, and glucose were quantified using colorimetric assays on a random-access Spectrum CCX analyzer (Abbott Diagnostics, Abbott Park, IL, USA). All examinations were performed in one accredited laboratory.

### 2.5. Blood Pressure (BP)

BP was assessed following the guidelines outlined in The Fourth Report on the Diagnosis, Evaluation, and Treatment of High Blood Pressure in Children and Adolescents [17]. Systolic blood pressure (SBP) and diastolic blood pressure (DBP) were recorded in the right arm utilizing a sphygmomanometer. Trained technicians conducted two separate measures prior to drawing blood samples, allowing for a rest period of 5 and 10 min while seated. The average of these two readings was utilized for subsequent analysis. 

In a sample of 89 students, the within-day technical error of measurement (σe) and the reliability (R) [18] for repeated measurements were determined as follows: SBP, 2.43 mm Hg; DBP, 2.52 mm Hg; reliability (R) coefficients were as follows: SBP, 0.96; DBP, 0.92.

### 2.6. MetS Risk Score

The syndrome’s definition and the threshold values for its individual components vary among different studies, with none focusing specifically on children and adolescents. Given that the central goal of the current study was to explore the aggregation of risk factors associated with low birth weight (LBW), a continuous metabolic syndrome risk score [19,20] was used. Each component (HDL-C, triglycerides, BP, insulin, blood glucose) was transformed into a *Z*-score using the formula *Z* = ([value − mean]/SD). If necessary, *Z*-scores were inverted by multiplying by −1 to reflect a higher metabolic risk with increasing values. The *Z*-scores for systolic and diastolic blood pressure were averaged to create a single indicator. Subsequently, the *Z*-scores for the five criteria of metabolic syndrome were summed and divided by five to calculate an average metabolic syndrome risk score, consistent with methodologies employed in other epidemiological studies focusing on adolescents [1,21].

### 2.7. Daily Physical Activity (PA)

Participants engaged in a daily protocol [22] during three consecutive days (Thursday, Friday and Saturday). This procedure segmented each day into 96 intervals of 15 min. Participants were instructed to document all activities undertaken and to assess the intensity of the main activity conducted during each 15-min interval using a numeric scale from one to nine. Daily energy expenditure (EE) was assessed for each category (1 to 9) similarly to the original protocol from the Québec Family Study [22]. The overall daily energy expenditure was calculated for each of the three days. The intensity levels categorized as 6–9 (4.8–7.8 METs) indicated moderate-to-vigorous physical activity (MVPA) [23].

### 2.8. Cardio-Respiratory Fitness (CRF)

CRF was evaluated with the Progressive Aerobic Cardiovascular Endurance Run (PACER) test [24]. Participants were scored based on the total number of laps completed until they reached voluntary exhaustion. During the test, individuals ran back and forth between two lines spaced 20 m apart, adhering to a rhythm set by an audio CD that emitted signals at designated intervals. The starting running speed was set at 8.5 km/h for the first minute and increased by 0.5 km/h every subsequent minute. This assessment is recognized as a valid and reliable field method for estimating VO2max in adolescents [24]. Prior to commencing the test, students received a comprehensive explanation of the protocol. All assessments took place during physical education sessions in favorable dry weather conditions. Additionally, two master’s level students supervised each line 20-m apart to ensure adherence to the protocol and to provide encouragement and motivation for participants to exert maximum effort. At the conclusion of the test, all participants exhibited signs of significant exertion, including hyperpnea, facial flushing and grimacing, unsteady gait, and sweating.

### 2.9. Statistical Analyses

Descriptive statistics specific to each sex were computed for the variables of age, height, weight, BMI, birth weight, MVPA, CRF, and all metabolic variables. An a priori sample size was computed using the following criteria: expected mean difference of 1.0, standard deviation of 2.0, power of 95%, and error (alpha two-sided) of 0.05, with Bonferroni correction. This estimate required a minimum of 123 subjects in each group. A one-way analysis of covariance (ANCOVA) was employed to examine the influence of gender on the previously mentioned variables, while adjusting for chronological age. Following each ANCOVA, Bonferroni-corrected post hoc tests were conducted.

Prior to performing the analysis, the distributions of the clustering of metabolic risk factors, moderate-to-vigorous physical activity (MVPA), and cardiorespiratory fitness (CRF) scores were evaluated for normality and modified accordingly. Specifically, insulin, glucose, triglycerides, CRF, and MVPA underwent logarithmic transformation. This log transformation enhanced the normality of these variables, allowing them to be utilized as transformed variables in various analyses.

To evaluate the relations of low birth weight (LBW) with the clustered metabolic risk factors while controlling for potential confounding variables such as chronological age, body mass index (BMI), MVPA, and CRF, multiple linear regression analysis was employed. In the crude model (Model 1), LBW served as the only predictor of clustered metabolic risk. Subsequently, BMI and age were then included as possible confounders in Models 2 and 3, respectively. Then, MVPA was also added as potential confounder in Model 4. Finally, CRF was then included as a possible confounding variable (Model 5). SPSS 17.0 (SPSS Inc., Chicago, IL, USA) was used and significance was set at 5%.

## 3. Results

The Table 1 summarizes the features of the sample. Males and females exhibited no significant differences in BMI, triglycerides, and glucose. However, weight, height, and BP (systolic and diastolic) were, on average, significantly higher in males, but HDL cholesterol and insulin levels were significantly higher in girls. Boys revealed significantly higher both PA levels and CRF scores than girls.

### 3.1. Bivariate Associations

Among females, the clustered metabolic risk score exhibited a negative correlation with CRF (r = −0.13, *p* ≤ 0.05), and a positive association with weight (r = 0.52, *p* ≤ 0.01), height (r = 0.27, *p* ≤ 0.01), and BMI (r = 0.48, *p* ≤ 0.01). In addition, CRF was negatively related to BMI (r = −0.27, *p* ≤ 0.01) and weight (r = −0.21, *p* ≤ 0.01). The strength of those associations ranged from weak to moderate.

Among males, the clustered metabolic risk score exhibited a negative correlation with CRF (r = −0.16, *p* ≤ 0.05). Conversely, it showed positive associations with weight (r = 0.46, *p* ≤ 0.01), height (r = 0.21, *p* ≤ 0.01), and BMI (r = 0.48, *p* ≤ 0.01). In addition, CRF was inversely related to LDL (r = −0.30, *p* ≤ 0.01) and total cholesterol (r = −0.35, *p* ≤ 0.01), as well as with triglycerides (r = −0.25, *p* ≤ 0.01), and positively correlated to MVPA (r = 0.34, *p* ≤ 0.01). Furthermore, male birth weight was inversely correlated to LDL (r = −0.19, *p* ≤ 0.05) and total cholesterol (r = −0.19, *p* ≤ 0.05), as well as to triglycerides (r = −0.16, *p* ≤ 0.05). As expected, the magnitude of those relationships ranged from weak to moderate.

### 3.2. Relationship of LBW with the Clustered Metabolic Risk Score

The outcomes of the regression analyses for boys and girls are presented in Table 2 and Table 3, respectively. 

In boys, LBW revealed a significant association with the clustered metabolic risk *Z*-score after controlling for confounding variables (β = −0.01; 95% CI, −0.01–0.00) Furthermore, in the final model, body mass index (BMI) emerged as another significant predictor of the clustered metabolic risk among males (β = 0.26; 95% CI, 0.16–0.36) (Table 3).

Among females, the crude model showed a significant correlation between LBW and the clustered metabolic risk *Z*-score. However, after controlling for possible confounding factors in Model 3, this relationship lost its significance in Model 4, which contrasts with the results found among males. Of relevance, in the final model, BMI (β = 0.02; 95% CI, 0.14–0.30) and MVPA (β = −0.01; 95% CI, −0.10–−0.01) were the unique significant predictors of clustered metabolic risk in girls (Table 2).

## 4. Discussion

Pediatric obesity continues to be a significant issue in global health, as projections indicate that the number of youths between the ages of 5 and 19 years who are classified as obese could reach 254 million by the year 2030. Furthermore, excess body fat is a critical indicator of potential metabolic and cardiovascular risks among young individuals. The present study revealed that LBW was found to be independently linked to the aggregation of MRF in boys. In fact, the transition from a hypo-trophic intrauterine environment, which can result in adipocyte hyperplasia, increases the possibility of adipose hypertrophy in a postnatal environment with access to nutrients [11]. As previously reported, that mechanism can induce excessive weight gain and therefore contribute to the development of non-communicable diseases such as obesity, hypertension, and heart disease later in life [12,13]. Interestingly, similar results were found among European [10,25], Australian [26], and Asian [4] youth. Indeed, LBW youth face a heightened risk of experiencing growth deficiencies during early childhood. This can potentially result in becoming overweight, which may subsequently lead to changes in blood pressure and other chronic cardiometabolic issues as they progress from childhood and adolescence to adulthood [10]. Consequently, it is essential to implement early and continuous clinical monitoring for individuals born preterm to facilitate the prompt identification and management of conditions that may contribute to the onset of MetS and CVD in later life.

Girls in the current research did not evidence any substantial association between LBW and the clustered metabolic risk *Z*-score. The results of the present study diverge from earlier research, which has identified female sex as a risk factor linked to the relationship between LBW and negative health outcomes including cardiometabolic issues, glucose metabolism disorders, and variations in body composition [3,10]. Studies have also pointed out that this phenomenon occurred because of heightened blood pressure responses to psychosocial stressors, which were significantly more pronounced in females compared to males. Additionally, the influence of unadjusted confounding variables, including maternal or genetic factors, may also play a role [4,27,28]. The contrasting findings among female adolescents in the present study highlight the complex dynamics of the interactions occurring within the uterus between a mother and her fetus, which appear to disrupt organic homeostasis in distinct ways for male and female offspring.

Furthermore, the differences in results across studies might be attributed to the cut-off criteria employed to express MRF, since a widely accepted and all-encompassing definition of MetS for children and adolescents has not yet been established. For example, findings for the Brazilian adolescents revealed substantial variation in MetS prevalences [29,30] which require careful interpretation, even from longitudinal approaches that are limited and inconsistent [31]. Indeed, substantial heterogeneity between studies is also observed, which can be attributed to encompassing participants whose birth weights fall at both the lower and upper ends of the distribution. This variability may help to clarify some of the conflicting results observed in the literature. Furthermore, the main research has been focused on BW and postnatal weight gain, often neglecting to consider growth in length. Actually, weight for length at earlier ages of life could serve as a more accurate predictor of being overweight or obese in the future than weight alone [5]. Therefore, experimental and longitudinal studies are indispensable for establishing conclusions regarding the causal impact of biological factors, including maturation, combined with behavioural factors, on cardio-metabolic risk by sex.

Consistent results were observed in the final regression model of the present study, which pointed out BMI as the unique significant predictor of clustered metabolic risk of both boys and girls. Actually, being overweight or obese has been consistently recognized by the WHO as one of the five primary risk factors for non-communicable disease (NCD), mainly diabetes mellitus and cardiovascular disease (CVD) [32]. Moreover, insulin resistance is related to inflammatory mechanisms, leading to significant evidence of its correlation with obesity, particularly in research aimed at uncovering the initial stages of this pathological condition. Thus, insulin resistance could be either a contributing factor to or a consequence of obesity, as adipose tissue generates pro-inflammatory cytokines and adipokines that directly influence insulin metabolism [33]. Furthermore, the encouragement of high-energy, aggressive feeding practices aimed at achieving swift growth during early childhood has an important role in the onset of obesity and irregular fat distribution. Previous research [3,34] has revealed the inadequate use of growth recovery methods may convert the saving environment into one that promotes insulin resistance, potentially resulting in long-term obesity development.

Another source of variation among female adolescents of the present study was some lifestyle features, such as the daily level of PA. Recent research suggests that habitual PA plays a moderating role in the relationship with adiposity, insulin resistance and sensitivity, LDL-c, and adiponectin [35], as well as that interventions that included exercise were successful in enhancing body fat reduction and lowering metabolic risk [36]. Besides, the findings of the current study revealed that more active females had considerably lower metabolic risk than their less active counterparts. Alongside the biological and behavioral dimensions contributing to the noted disparities between sexes, it is plausible that the observed sex difference in the relationship of MVPA with the clustered metabolic risk may be partially attributed to the accuracy of assessment methods, as subjective tools tend to be significantly less reliable for measuring high-intensity levels of regular PA. Despite recent developments in algorithms that incorporate signals from multiple sensors and reliability improvements in self-reporting methods that have shown higher efficacy in particular types of assessments of adolescents’ physical activity [37], most of their daily activities are intermittent and spontaneous, and therefore, they are probably more difficult to assess than structured and/or occupational activities [38]. Thus, it is advisable for future studies involving adolescents to employ research strategies that use a multi-method design, integrating both quantitative assessment measures—such as accelerometers, heart rate monitors, and other objective tools—and qualitative data regarding PA as a multidimensional construct.

In summary, despite the very interesting results of the present study, which incorporated a remarkable combined approach of biological attributes and behavioural factors associated with metabolic risk in adolescents, caution should be exercised when comparing the observed associations in Brazilian adolescents to those found in other populations. Accordingly, the current research has some limitations that ought to be acknowledged. Firstly, the causal association of LBW with increased metabolic risk cannot be deduced from studies with a cross-sectional analytical approach; therefore, longitudinal studies are required to draw inferences about the aforementioned relationship to identify vulnerable groups at risk of cardiovascular disease in the future. Moreover, the study protocol did not include an evaluation of biological maturity status; while chronological age was accounted for in the analyses, this adjustment might not be adequate, as biological maturity can influence insulin levels in adolescents. Therefore, it is essential to consider biological maturity in future research. Furthermore, the use of self-reported tools for habitual PA assessment presents significant challenges, and it certainly requires careful consideration and the implementation of standardized procedures to reduce the likelihood of measurement errors.

## 5. Conclusions

LBW was identified as having an independent association with an increased metabolic risk among male Brazilian adolescents. Interventions aimed at improving metabolic health in youth should target the promotion of MVPA, especially among females. Experimental and longitudinal research is especially required to establish reliable inferences about the etiologic impact of biological and behavioural factors on cardio-metabolic risk.

## Figures and Tables

**Table 1:** Descriptive characteristics (mean and standard deviation) of students.

Variable	All	Males	Females	η^2^
		(n = 229)	(n = 262)	
Age, years	15.1 ± 1.1	15.2 ± 1.2	15.2 ± 1.1	0.00
Weight (kg)	57.5 ± 12.1	61.0 ± 13.0	54.3 ± 10.3 **	0.08
Height (m)	164.5 ± 9.7	170.0 ± 10.1	159.6 ± 6.1 **	0.29
BMI (kg/m^2^)	21.12 ± 3.33	20.98 ± 3.19	21.25 ± 3.45	0.00
Birth weight (g)	3193 ± 571	3278 ± 580	3119 ± 553 **	0.02
Insulin ^a^ (pmol/L)	5.88 ± 3.48	4.76 ± 3.80	6.86 ± 2.83 **	0.09
Glucose ^a^ (mmol/L)	96.06 ± 13.23	95.59 ± 10.84	96.47 ± 15.02	0.00
Triglycerides ^a^ (mmol/L)	85.62 ± 36.26	85.00 ± 41.20	86.16 ± 31.35	0.00
HDL cholesterol (mmol/L)	44.10 ± 10.34	42.78 ± 9.03	45.25 ± 11.25 **	0.01
Systolic BP (mmHg)	104.46 ± 12.43	107.60 ± 12.55	101.71 ± 11.67 **	0.06
Diastolic BP (mmHg)	69.97 ± 9.55	71.60 ± 9.61	68.55 ± 9.28 **	0.03
Metabolic syndrome (*Z*-score)	0.41 ± 2.63	0.46 ± 2.72	0.37 ± 2.55	0.00
CRF ^a,b^ (#)	35.4 ± 15.5	63.1 ± 22.6	30.4 ± 11.2 **	0.47
MVPA ^a^ (minutes)	78.0 ± 82.0	110.6 ± 86.4	49.6 ± 66.1 **	0.14

** *p* < 0.01; ^a^ Log-transformed values were used in the analysis; ^b^ Adjusted for age and gender; BP (blood pressure); MVPA (moderate-to-vigorous physical activity).

**Table 2:** Prediction of the metabolic syndrome *Z*-score in males between the ages of 14 and 17.

					Metabolic Syndrome *Z*-Score				
Model		R^2^	Adjusted R^2^	Predictor	Unstandardized Coefficients		95% CI for Beta		Standardized Beta Coefficient
					Beta	St. Error	Lower	Upper	
	F_(1,229)_ = 4.067 (*p* < 0.05)	1.8%	1.3%	LBW	0.00	0.00	0.00	0.00	0.13
	F_(1,228)_ = 30.170 (*p* < 0.01)	13.3%	12.5%	LBW	−0.01	0.00	−0.002	−0.001	−0.30
				BMI	0.19	0.04	0.12	0.26	0.34
MALES (n = 229)	F_(1,227)_ = 4.899 (*p* < 0.05)	15.1%	14.0%	LBW	−0.001	0.00	−0.001	0.00	−0.20
				BMI	0.27	0.05	0.17	0.36	0.34
				Age	−0.16	0.07	−0.31	−0.02	−0.14
	F_(1,226)_ = 0.012 (*p* = 0.91)	15.1%	13.6%	LBW	−0.001	0.00	−0.001	0.00	−0.20
				BMI	0.27	0.05	0.17	0.36	0.34
				Age	−0.16	0.08	−0.31	−0.02	−0.14
	F_(1,225)_ = 1.748 (*p* = 0.19)	15.8%	13.1%	LBW	−0.001	0.00	−0.001	0.00	−0.20
				BMI	0.26	0.05	0.16	0.36	0.32

Model 1 = unadjusted; Model 2 = adjusted for BMI (body mass index); Model 3 = model 2 + chronological age; Model 4 = model 3 + adjusted for MVPA (moderate-to-vigorous physical activity); Model 5 = model 4 + adjusted for CRF (cardiorespiratory fitness). LBW (low birth weight).

**Table 3:** Prediction of the metabolic syndrome *Z*-score in females between the ages of 14 and 17.

					Metabolic Syndrome *Z*-Score				
Model		R^2^	Adjusted R^2^	Predictor	Unstandardized Coefficients		95% CI for Beta		Standardized Beta Coefficient
					Beta	St. Error	Lower	Upper	
	F_(1,262)_ = 5.307 (*p* < 0.05)	2.0%	1.6%	LBW	0.00	0.00	0.00	0.00	−0.14
	F_(1,261)_ = 20.784 (*p* < 0.01)	9.2%	8.5%	LBW	−0.001	0.00	−0.001	0.00	−0.23
				BMI	0.14	0.03	0.08	0.21	0.27
FEMALES (n = 262)	F_(1,260)_ = 10.788 (*p* < 0.01)	12.9%	11.9%	BMI	0.23	0.04	0.15	0.31	0.33
				AGE	−0.22	0.07	−0.35	−0.09	−0.19
	F_(1,259)_ = 7.205 (*p* < 0.01)	15.2%	13.9%	BMI	0.22	0.04	0.14	0.30	0.32
				Age	−0.19	0.07	−0.32	−0.06	−0.17
				MVPA	−0.01	0.02	−0.01	−0.002	−0.15
	F_(1,258)_ = 0.795 (*p* = 0.37)	15.5%	13.9%	BMI	0.22	0.04	0.14	0.30	0.30
				Age	−0.16	0.08	−0.31	−0.01	−0.12
				MVPA	−0.01	0.02	−0.10	−0.01	−0.15

Model 1 = unadjusted; Model 2 = adjusted for BMI (body mass index); Model 3 = model 2 + chronological age; Model 4 = model 3 + adjusted for MVPA (moderate-to-vigorous physical activity); Model 5 = model 4 + adjusted for CRF (cardiorespiratory fitness). LBW (low birth weight).

## Data Availability

The data presented in this study are available on request from the corresponding author due to privacy and ethical restrictions.
